# Supplementary material for: Meta-Analysis and Gene Set Enrichment Relative to ER Status Reveal Elevated Activity of MYC and E2F in the “Basal” Breast Cancer Subgroup
Source: PLoS One. 2009 Mar 9;4(3):e4710. doi: 10.1371/journal.pone.0004710 (PMC2650420; doi:10.1371/journal.pone.0004710)
Supplement: Dataset S1 — GSEA results from first screen. The file contains a zipped archive of the HTML results of the second GSEA screen. Reports for enriched or depleted datasets may be accessed from the “index.html” link within the my_analysis.GseaPreranked.1219975950625.rpt.zip ZIP archive. Enrichment plots are available for each of the 50 most enriched and depleted gene sets. (13.32 MB ZIP) [file pone.0004710.s001.zip › my_analysis.GseaPreranked.1219975950625.rpt.zip!//BRCA_ER_NEG.html]

Details for gene set BRCA\_ER\_NEG[GSEA]

|  || Dataset | zSco.ERneg.to.ERpos\_collapsed |
| Phenotype | NoPhenotypeAvailable |
| Upregulated in class | na\_pos |
| GeneSet | BRCA\_ER\_NEG |
| Enrichment Score (ES) | 0.72467095 |
| Normalized Enrichment Score (NES) | 3.6675255 |
| Nominal p-value | 0.0 |
| FDR q-value | 0.0 |
| FWER p-Value | 0.0 |
Table: GSEA Results Summary

  

Fig 1: Enrichment plot: BRCA\_ER\_NEG      
 Profile of the Running ES Score & Positions of GeneSet Members on the Rank Ordered List

  

| PROBE | GENE SYMBOL | GENE\_TITLE | RANK IN GENE LIST | RANK METRIC SCORE | RUNNING ES | CORE ENRICHMENT || 1 | CTSL2 |  |  | 1 | 7.773 | 0.0027 | Yes |
| 2 | MMP7 |  |  | 2 | 7.609 | 0.0054 | Yes |
| 3 | UQCRH |  |  | 5 | 7.406 | 0.0078 | Yes |
| 4 | PADI2 |  |  | 7 | 7.278 | 0.0103 | Yes |
| 5 | TES |  |  | 8 | 7.200 | 0.0129 | Yes |
| 6 | CDC20 |  |  | 9 | 7.151 | 0.0154 | Yes |
| 7 | SLC43A3 |  |  | 10 | 7.126 | 0.0180 | Yes |
| 8 | TTK |  |  | 11 | 7.059 | 0.0205 | Yes |
| 9 | SRPK1 |  |  | 12 | 6.990 | 0.0229 | Yes |
| 10 | FAT |  |  | 13 | 6.974 | 0.0254 | Yes |
| 11 | BCL11A |  |  | 14 | 6.962 | 0.0279 | Yes |
| 12 | LPIN1 |  |  | 15 | 6.951 | 0.0304 | Yes |
| 13 | BBOX1 |  |  | 16 | 6.950 | 0.0328 | Yes |
| 14 | PDHA1 |  |  | 17 | 6.929 | 0.0353 | Yes |
| 15 | VGLL1 |  |  | 18 | 6.865 | 0.0377 | Yes |
| 16 | MSN |  |  | 20 | 6.766 | 0.0400 | Yes |
| 17 | FOXM1 |  |  | 21 | 6.760 | 0.0424 | Yes |
| 18 | IFRD1 |  |  | 22 | 6.733 | 0.0448 | Yes |
| 19 | LMO4 |  |  | 23 | 6.717 | 0.0472 | Yes |
| 20 | PRKX |  |  | 26 | 6.673 | 0.0494 | Yes |
| 21 | FAM60A |  |  | 27 | 6.642 | 0.0518 | Yes |
| 22 | MCM5 |  |  | 31 | 6.533 | 0.0538 | Yes |
| 23 | HDAC2 |  |  | 32 | 6.511 | 0.0562 | Yes |
| 24 | ORC6L |  |  | 33 | 6.507 | 0.0585 | Yes |
| 25 | GLS |  |  | 35 | 6.500 | 0.0607 | Yes |
| 26 | TGFA |  |  | 37 | 6.489 | 0.0629 | Yes |
| 27 | CRYAB |  |  | 39 | 6.465 | 0.0651 | Yes |
| 28 | RARRES1 |  |  | 40 | 6.454 | 0.0674 | Yes |
| 29 | BUB1 |  |  | 41 | 6.445 | 0.0697 | Yes |
| 30 | GSTP1 |  |  | 42 | 6.388 | 0.0720 | Yes |
| 31 | MRAS |  |  | 43 | 6.382 | 0.0742 | Yes |
| 32 | SFRP1 |  |  | 44 | 6.369 | 0.0765 | Yes |
| 33 | FSCN1 |  |  | 46 | 6.343 | 0.0787 | Yes |
| 34 | PKP1 |  |  | 48 | 6.341 | 0.0808 | Yes |
| 35 | PTX3 |  |  | 49 | 6.328 | 0.0831 | Yes |
| 36 | TMSL8 |  |  | 50 | 6.319 | 0.0853 | Yes |
| 37 | CENPA |  |  | 51 | 6.315 | 0.0876 | Yes |
| 38 | CCNA2 |  |  | 52 | 6.297 | 0.0898 | Yes |
| 39 | PFDN2 |  |  | 53 | 6.292 | 0.0920 | Yes |
| 40 | KCNN4 |  |  | 54 | 6.289 | 0.0943 | Yes |
| 41 | PROM1 |  |  | 55 | 6.272 | 0.0965 | Yes |
| 42 | BLM |  |  | 56 | 6.255 | 0.0987 | Yes |
| 43 | PRNP |  |  | 58 | 6.204 | 0.1008 | Yes |
| 44 | NASP |  |  | 59 | 6.201 | 0.1030 | Yes |
| 45 | STIL |  |  | 60 | 6.200 | 0.1052 | Yes |
| 46 | DKFZP762E1312 |  |  | 61 | 6.161 | 0.1074 | Yes |
| 47 | AGTPBP1 |  |  | 62 | 6.128 | 0.1096 | Yes |
| 48 | CDK2AP1 |  |  | 63 | 6.118 | 0.1118 | Yes |
| 49 | LYN |  |  | 64 | 6.117 | 0.1139 | Yes |
| 50 | SLC6A14 |  |  | 70 | 6.065 | 0.1157 | Yes |
| 51 | NMB |  |  | 71 | 6.063 | 0.1178 | Yes |
| 52 | CEBPG |  |  | 72 | 6.061 | 0.1200 | Yes |
| 53 | AURKB |  |  | 76 | 6.038 | 0.1219 | Yes |
| 54 | PIM1 |  |  | 78 | 6.025 | 0.1239 | Yes |
| 55 | FABP7 |  |  | 79 | 6.019 | 0.1261 | Yes |
| 56 | LDHB |  |  | 82 | 6.005 | 0.1280 | Yes |
| 57 | KLF5 |  |  | 87 | 5.966 | 0.1298 | Yes |
| 58 | STAC |  |  | 90 | 5.955 | 0.1318 | Yes |
| 59 | YES1 |  |  | 91 | 5.947 | 0.1339 | Yes |
| 60 | ODC1 |  |  | 94 | 5.931 | 0.1358 | Yes |
| 61 | TRIP13 |  |  | 96 | 5.890 | 0.1378 | Yes |
| 62 | FANCA |  |  | 99 | 5.866 | 0.1398 | Yes |
| 63 | PLA2G4A |  |  | 100 | 5.860 | 0.1418 | Yes |
| 64 | NFIB |  |  | 101 | 5.857 | 0.1439 | Yes |
| 65 | BTG3 |  |  | 102 | 5.850 | 0.1460 | Yes |
| 66 | GPRC5B |  |  | 103 | 5.845 | 0.1481 | Yes |
| 67 | RUNX3 |  |  | 105 | 5.835 | 0.1501 | Yes |
| 68 | SOX10 |  |  | 107 | 5.831 | 0.1521 | Yes |
| 69 | CDC45L |  |  | 108 | 5.824 | 0.1541 | Yes |
| 70 | LRP12 |  |  | 109 | 5.815 | 0.1562 | Yes |
| 71 | RBM28 |  |  | 116 | 5.736 | 0.1577 | Yes |
| 72 | TRIM29 |  |  | 119 | 5.731 | 0.1596 | Yes |
| 73 | TRIM2 |  |  | 121 | 5.724 | 0.1616 | Yes |
| 74 | RRAGD |  |  | 122 | 5.723 | 0.1636 | Yes |
| 75 | CTPS |  |  | 123 | 5.706 | 0.1656 | Yes |
| 76 | KIF2C |  |  | 124 | 5.703 | 0.1676 | Yes |
| 77 | IL32 |  |  | 125 | 5.701 | 0.1697 | Yes |
| 78 | ST8SIA1 |  |  | 130 | 5.690 | 0.1714 | Yes |
| 79 | UCK2 |  |  | 132 | 5.685 | 0.1733 | Yes |
| 80 | ARL4C |  |  | 134 | 5.675 | 0.1752 | Yes |
| 81 | KCNK5 |  |  | 136 | 5.658 | 0.1772 | Yes |
| 82 | FAM64A |  |  | 137 | 5.658 | 0.1792 | Yes |
| 83 | TEX10 |  |  | 139 | 5.644 | 0.1811 | Yes |
| 84 | SLPI |  |  | 140 | 5.641 | 0.1831 | Yes |
| 85 | SNRPG |  |  | 142 | 5.628 | 0.1850 | Yes |
| 86 | DAPK1 |  |  | 144 | 5.606 | 0.1869 | Yes |
| 87 | GOLT1B |  |  | 145 | 5.605 | 0.1889 | Yes |
| 88 | RAD54L |  |  | 146 | 5.602 | 0.1909 | Yes |
| 89 | TTYH1 |  |  | 147 | 5.596 | 0.1929 | Yes |
| 90 | TMEM123 |  |  | 149 | 5.586 | 0.1948 | Yes |
| 91 | RIPK4 |  |  | 150 | 5.576 | 0.1967 | Yes |
| 92 | NOL1 |  |  | 152 | 5.558 | 0.1986 | Yes |
| 93 | S100A2 |  |  | 153 | 5.556 | 0.2006 | Yes |
| 94 | CXCL1 |  |  | 154 | 5.553 | 0.2026 | Yes |
| 95 | KIFC1 |  |  | 156 | 5.525 | 0.2045 | Yes |
| 96 | YBX1 |  |  | 157 | 5.523 | 0.2064 | Yes |
| 97 | GGH |  |  | 161 | 5.504 | 0.2081 | Yes |
| 98 | LRP8 |  |  | 163 | 5.498 | 0.2100 | Yes |
| 99 | DSC2 |  |  | 166 | 5.483 | 0.2118 | Yes |
| 100 | ZNF259 |  |  | 167 | 5.482 | 0.2137 | Yes |
| 101 | CX3CL1 |  |  | 169 | 5.477 | 0.2156 | Yes |
| 102 | CIB2 |  |  | 170 | 5.476 | 0.2175 | Yes |
| 103 | IVNS1ABP |  |  | 172 | 5.467 | 0.2194 | Yes |
| 104 | PSME4 |  |  | 175 | 5.460 | 0.2212 | Yes |
| 105 | PDSS1 |  |  | 179 | 5.440 | 0.2229 | Yes |
| 106 | MCM4 |  |  | 180 | 5.436 | 0.2248 | Yes |
| 107 | MELK |  |  | 181 | 5.424 | 0.2267 | Yes |
| 108 | DEFB1 |  |  | 183 | 5.405 | 0.2286 | Yes |
| 109 | DEK |  |  | 190 | 5.364 | 0.2300 | Yes |
| 110 | CAPN6 |  |  | 191 | 5.356 | 0.2319 | Yes |
| 111 | CDCA8 |  |  | 194 | 5.328 | 0.2336 | Yes |
| 112 | D21S2056E |  |  | 195 | 5.326 | 0.2355 | Yes |
| 113 | PAK1IP1 |  |  | 197 | 5.319 | 0.2373 | Yes |
| 114 | MCM6 |  |  | 198 | 5.317 | 0.2392 | Yes |
| 115 | DSC3 |  |  | 200 | 5.315 | 0.2410 | Yes |
| 116 | MTHFD2 |  |  | 205 | 5.307 | 0.2426 | Yes |
| 117 | GMNN |  |  | 206 | 5.302 | 0.2444 | Yes |
| 118 | RDX |  |  | 207 | 5.298 | 0.2463 | Yes |
| 119 | DSCR2 |  |  | 208 | 5.293 | 0.2482 | Yes |
| 120 | NUP93 |  |  | 210 | 5.281 | 0.2500 | Yes |
| 121 | EXO1 |  |  | 218 | 5.239 | 0.2513 | Yes |
| 122 | KIF14 |  |  | 221 | 5.215 | 0.2530 | Yes |
| 123 | USP1 |  |  | 222 | 5.212 | 0.2548 | Yes |
| 124 | KARS |  |  | 223 | 5.209 | 0.2567 | Yes |
| 125 | PTTG1 |  |  | 225 | 5.206 | 0.2584 | Yes |
| 126 | TEAD4 |  |  | 226 | 5.204 | 0.2603 | Yes |
| 127 | PLD1 |  |  | 228 | 5.200 | 0.2620 | Yes |
| 128 | MAGOH |  |  | 230 | 5.186 | 0.2638 | Yes |
| 129 | TPX2 |  |  | 231 | 5.180 | 0.2656 | Yes |
| 130 | REXO2 |  |  | 234 | 5.170 | 0.2673 | Yes |
| 131 | LRRC8D |  |  | 237 | 5.158 | 0.2690 | Yes |
| 132 | GFOD1 |  |  | 238 | 5.149 | 0.2708 | Yes |
| 133 | C11ORF75 |  |  | 242 | 5.132 | 0.2724 | Yes |
| 134 | HMGA1 |  |  | 244 | 5.125 | 0.2741 | Yes |
| 135 | TP53BP2 |  |  | 245 | 5.119 | 0.2759 | Yes |
| 136 | LAMP3 |  |  | 247 | 5.108 | 0.2777 | Yes |
| 137 | ATP1B3 |  |  | 249 | 5.095 | 0.2794 | Yes |
| 138 | SOX11 |  |  | 250 | 5.094 | 0.2812 | Yes |
| 139 | EHBP1 |  |  | 251 | 5.094 | 0.2830 | Yes |
| 140 | KNTC2 |  |  | 256 | 5.074 | 0.2845 | Yes |
| 141 | TUBB6 |  |  | 264 | 5.044 | 0.2857 | Yes |
| 142 | PDCD5 |  |  | 270 | 5.025 | 0.2871 | Yes |
| 143 | EGFL6 |  |  | 276 | 5.005 | 0.2885 | Yes |
| 144 | PLCG2 |  |  | 277 | 5.004 | 0.2903 | Yes |
| 145 | CEP55 |  |  | 279 | 4.995 | 0.2919 | Yes |
| 146 | NMT2 |  |  | 282 | 4.991 | 0.2936 | Yes |
| 147 | OXCT1 |  |  | 283 | 4.991 | 0.2953 | Yes |
| 148 | NPC1 |  |  | 284 | 4.988 | 0.2971 | Yes |
| 149 | ANGPTL4 |  |  | 285 | 4.987 | 0.2989 | Yes |
| 150 | MALL |  |  | 286 | 4.987 | 0.3006 | Yes |
| 151 | MCM7 |  |  | 289 | 4.970 | 0.3022 | Yes |
| 152 | TMEM158 |  |  | 290 | 4.964 | 0.3040 | Yes |
| 153 | GPSM2 |  |  | 291 | 4.963 | 0.3058 | Yes |
| 154 | CCDC93 |  |  | 294 | 4.960 | 0.3074 | Yes |
| 155 | CORO1C |  |  | 299 | 4.934 | 0.3088 | Yes |
| 156 | CTSC |  |  | 300 | 4.932 | 0.3105 | Yes |
| 157 | CHI3L1 |  |  | 301 | 4.931 | 0.3123 | Yes |
| 158 | TNF |  |  | 302 | 4.930 | 0.3140 | Yes |
| 159 | NCK1 |  |  | 305 | 4.928 | 0.3156 | Yes |
| 160 | STMN1 |  |  | 310 | 4.907 | 0.3170 | Yes |
| 161 | EZH2 |  |  | 312 | 4.888 | 0.3187 | Yes |
| 162 | DCPS |  |  | 313 | 4.884 | 0.3204 | Yes |
| 163 | CLIC4 |  |  | 314 | 4.881 | 0.3222 | Yes |
| 164 | CALU |  |  | 315 | 4.878 | 0.3239 | Yes |
| 165 | KIF20A |  |  | 318 | 4.874 | 0.3255 | Yes |
| 166 | TTLL4 |  |  | 319 | 4.873 | 0.3272 | Yes |
| 167 | IFNAR2 |  |  | 325 | 4.864 | 0.3285 | Yes |
| 168 | MCCC1 |  |  | 326 | 4.861 | 0.3302 | Yes |
| 169 | FGFBP1 |  |  | 327 | 4.860 | 0.3320 | Yes |
| 170 | C1ORF121 |  |  | 328 | 4.860 | 0.3337 | Yes |
| 171 | ANXA1 |  |  | 332 | 4.853 | 0.3352 | Yes |
| 172 | CSDA |  |  | 337 | 4.835 | 0.3366 | Yes |
| 173 | CDC25B |  |  | 340 | 4.826 | 0.3381 | Yes |
| 174 | TFCP2L1 |  |  | 342 | 4.825 | 0.3397 | Yes |
| 175 | EGFR |  |  | 344 | 4.822 | 0.3414 | Yes |
| 176 | NCAPH |  |  | 348 | 4.799 | 0.3428 | Yes |
| 177 | DSG3 |  |  | 350 | 4.785 | 0.3445 | Yes |
| 178 | CENPN |  |  | 352 | 4.783 | 0.3461 | Yes |
| 179 | ASPM |  |  | 359 | 4.767 | 0.3473 | Yes |
| 180 | SCRG1 |  |  | 362 | 4.762 | 0.3488 | Yes |
| 181 | RND3 |  |  | 364 | 4.755 | 0.3504 | Yes |
| 182 | RHEB |  |  | 366 | 4.750 | 0.3520 | Yes |
| 183 | SNX3 |  |  | 367 | 4.750 | 0.3537 | Yes |
| 184 | RFC4 |  |  | 370 | 4.741 | 0.3552 | Yes |
| 185 | NOTCH1 |  |  | 374 | 4.737 | 0.3567 | Yes |
| 186 | C10ORF7 |  |  | 375 | 4.736 | 0.3583 | Yes |
| 187 | C10ORF38 |  |  | 377 | 4.733 | 0.3599 | Yes |
| 188 | MFGE8 |  |  | 384 | 4.720 | 0.3611 | Yes |
| 189 | CDH19 |  |  | 385 | 4.720 | 0.3628 | Yes |
| 190 | UGT8 |  |  | 387 | 4.707 | 0.3644 | Yes |
| 191 | UBE2E3 |  |  | 392 | 4.698 | 0.3657 | Yes |
| 192 | RNASEH1 |  |  | 396 | 4.692 | 0.3672 | Yes |
| 193 | DYSF |  |  | 399 | 4.687 | 0.3687 | Yes |
| 194 | JRKL |  |  | 400 | 4.686 | 0.3703 | Yes |
| 195 | ATP11B |  |  | 401 | 4.682 | 0.3720 | Yes |
| 196 | SYNCRIP |  |  | 402 | 4.679 | 0.3737 | Yes |
| 197 | C16ORF80 |  |  | 403 | 4.674 | 0.3753 | Yes |
| 198 | CREB3L2 |  |  | 404 | 4.668 | 0.3770 | Yes |
| 199 | DEPDC1 |  |  | 406 | 4.663 | 0.3785 | Yes |
| 200 | MID1 |  |  | 411 | 4.653 | 0.3799 | Yes |
| 201 | GABRP |  |  | 412 | 4.649 | 0.3815 | Yes |
| 202 | CKS1B |  |  | 416 | 4.638 | 0.3829 | Yes |
| 203 | MYBL2 |  |  | 418 | 4.629 | 0.3845 | Yes |
| 204 | AKR1B1 |  |  | 420 | 4.620 | 0.3861 | Yes |
| 205 | CSTB |  |  | 427 | 4.595 | 0.3872 | Yes |
| 206 | CCNJ |  |  | 430 | 4.590 | 0.3887 | Yes |
| 207 | CCNB2 |  |  | 431 | 4.589 | 0.3903 | Yes |
| 208 | ALDH1A3 |  |  | 434 | 4.575 | 0.3918 | Yes |
| 209 | IL27RA |  |  | 436 | 4.574 | 0.3933 | Yes |
| 210 | C11ORF41 |  |  | 438 | 4.563 | 0.3948 | Yes |
| 211 | SLC2A6 |  |  | 441 | 4.551 | 0.3963 | Yes |
| 212 | C2ORF4 |  |  | 445 | 4.532 | 0.3977 | Yes |
| 213 | OPTN |  |  | 446 | 4.530 | 0.3993 | Yes |
| 214 | C16ORF61 |  |  | 447 | 4.520 | 0.4009 | Yes |
| 215 | SMC4 |  |  | 448 | 4.519 | 0.4025 | Yes |
| 216 | MPZL1 |  |  | 449 | 4.518 | 0.4041 | Yes |
| 217 | ART3 |  |  | 450 | 4.517 | 0.4057 | Yes |
| 218 | AURKA |  |  | 460 | 4.483 | 0.4065 | Yes |
| 219 | ROR1 |  |  | 463 | 4.481 | 0.4080 | Yes |
| 220 | HRK |  |  | 464 | 4.475 | 0.4096 | Yes |
| 221 | ENO1 |  |  | 465 | 4.475 | 0.4112 | Yes |
| 222 | GZMB |  |  | 466 | 4.470 | 0.4127 | Yes |
| 223 | UCHL1 |  |  | 472 | 4.462 | 0.4139 | Yes |
| 224 | SLC5A6 |  |  | 474 | 4.453 | 0.4154 | Yes |
| 225 | MAD2L1 |  |  | 477 | 4.446 | 0.4168 | Yes |
| 226 | CHAF1B |  |  | 479 | 4.411 | 0.4183 | Yes |
| 227 | DMD |  |  | 481 | 4.406 | 0.4198 | Yes |
| 228 | CDH3 |  |  | 484 | 4.397 | 0.4212 | Yes |
| 229 | SRD5A1 |  |  | 486 | 4.391 | 0.4227 | Yes |
| 230 | ICAM1 |  |  | 488 | 4.389 | 0.4242 | Yes |
| 231 | FLJ20186 |  |  | 489 | 4.372 | 0.4257 | Yes |
| 232 | PARVB |  |  | 490 | 4.372 | 0.4273 | Yes |
| 233 | FAM107A |  |  | 492 | 4.366 | 0.4287 | Yes |
| 234 | EPHA2 |  |  | 494 | 4.362 | 0.4302 | Yes |
| 235 | KHDRBS3 |  |  | 498 | 4.353 | 0.4315 | Yes |
| 236 | TNFRSF21 |  |  | 504 | 4.342 | 0.4326 | Yes |
| 237 | MAPRE2 |  |  | 507 | 4.333 | 0.4340 | Yes |
| 238 | TNFAIP3 |  |  | 512 | 4.326 | 0.4352 | Yes |
| 239 | NMU |  |  | 520 | 4.311 | 0.4362 | Yes |
| 240 | DLG7 |  |  | 521 | 4.306 | 0.4377 | Yes |
| 241 | GMDS |  |  | 526 | 4.289 | 0.4389 | Yes |
| 242 | ZIC1 |  |  | 527 | 4.288 | 0.4404 | Yes |
| 243 | CHST2 |  |  | 530 | 4.281 | 0.4418 | Yes |
| 244 | PPP1CB |  |  | 533 | 4.274 | 0.4432 | Yes |
| 245 | CXADR |  |  | 538 | 4.262 | 0.4443 | Yes |
| 246 | SPIB |  |  | 541 | 4.255 | 0.4457 | Yes |
| 247 | PRC1 |  |  | 543 | 4.253 | 0.4471 | Yes |
| 248 | SFT2D2 |  |  | 544 | 4.253 | 0.4486 | Yes |
| 249 | SMOX |  |  | 546 | 4.252 | 0.4501 | Yes |
| 250 | SEC63 |  |  | 547 | 4.252 | 0.4516 | Yes |
| 251 | IL15RA |  |  | 549 | 4.239 | 0.4530 | Yes |
| 252 | ST14 |  |  | 550 | 4.236 | 0.4545 | Yes |
| 253 | IRAK1 |  |  | 553 | 4.230 | 0.4558 | Yes |
| 254 | DSCR1 |  |  | 555 | 4.225 | 0.4573 | Yes |
| 255 | NF2 |  |  | 558 | 4.220 | 0.4586 | Yes |
| 256 | PSMB2 |  |  | 569 | 4.204 | 0.4593 | Yes |
| 257 | NFE2L3 |  |  | 570 | 4.204 | 0.4608 | Yes |
| 258 | CYP39A1 |  |  | 575 | 4.197 | 0.4619 | Yes |
| 259 | WDR43 |  |  | 578 | 4.189 | 0.4633 | Yes |
| 260 | CCL13 |  |  | 585 | 4.180 | 0.4643 | Yes |
| 261 | SH2D2A |  |  | 588 | 4.175 | 0.4656 | Yes |
| 262 | PLAUR |  |  | 604 | 4.138 | 0.4659 | Yes |
| 263 | SPBC25 |  |  | 606 | 4.126 | 0.4672 | Yes |
| 264 | LIMK1 |  |  | 609 | 4.120 | 0.4685 | Yes |
| 265 | MYO10 |  |  | 611 | 4.111 | 0.4699 | Yes |
| 266 | MARCKS |  |  | 613 | 4.107 | 0.4713 | Yes |
| 267 | PIP3-E |  |  | 614 | 4.104 | 0.4727 | Yes |
| 268 | ASNS |  |  | 618 | 4.099 | 0.4740 | Yes |
| 269 | PSAT1 |  |  | 621 | 4.087 | 0.4752 | Yes |
| 270 | SHFM1 |  |  | 623 | 4.086 | 0.4766 | Yes |
| 271 | ACTG2 |  |  | 626 | 4.083 | 0.4779 | Yes |
| 272 | RRAS2 |  |  | 628 | 4.081 | 0.4793 | Yes |
| 273 | TLE1 |  |  | 629 | 4.080 | 0.4807 | Yes |
| 274 | EPHB3 |  |  | 635 | 4.064 | 0.4818 | Yes |
| 275 | TM4SF1 |  |  | 639 | 4.054 | 0.4830 | Yes |
| 276 | COL9A3 |  |  | 640 | 4.054 | 0.4844 | Yes |
| 277 | PAPD1 |  |  | 643 | 4.047 | 0.4857 | Yes |
| 278 | FUT3 |  |  | 652 | 4.024 | 0.4865 | Yes |
| 279 | DDX21 |  |  | 653 | 4.024 | 0.4879 | Yes |
| 280 | DDX11 |  |  | 654 | 4.021 | 0.4893 | Yes |
| 281 | PCOLCE2 |  |  | 657 | 4.019 | 0.4906 | Yes |
| 282 | AMD1 |  |  | 658 | 4.018 | 0.4920 | Yes |
| 283 | IMPA2 |  |  | 660 | 4.017 | 0.4933 | Yes |
| 284 | GPI |  |  | 663 | 4.015 | 0.4946 | Yes |
| 285 | UCHL3 |  |  | 667 | 4.000 | 0.4958 | Yes |
| 286 | PAPSS1 |  |  | 670 | 3.995 | 0.4970 | Yes |
| 287 | GEMIN4 |  |  | 672 | 3.988 | 0.4984 | Yes |
| 288 | EXT1 |  |  | 673 | 3.986 | 0.4998 | Yes |
| 289 | ACSL4 |  |  | 679 | 3.976 | 0.5008 | Yes |
| 290 | ME1 |  |  | 682 | 3.971 | 0.5021 | Yes |
| 291 | CCR1 |  |  | 689 | 3.952 | 0.5030 | Yes |
| 292 | IL8 |  |  | 692 | 3.946 | 0.5042 | Yes |
| 293 | ZBED4 |  |  | 695 | 3.942 | 0.5055 | Yes |
| 294 | MARCO |  |  | 699 | 3.934 | 0.5066 | Yes |
| 295 | CSRP2 |  |  | 706 | 3.916 | 0.5075 | Yes |
| 296 | KIF11 |  |  | 713 | 3.909 | 0.5084 | Yes |
| 297 | EDN1 |  |  | 715 | 3.905 | 0.5097 | Yes |
| 298 | TLE4 |  |  | 718 | 3.903 | 0.5109 | Yes |
| 299 | FZD9 |  |  | 719 | 3.900 | 0.5123 | Yes |
| 300 | ZNF532 |  |  | 726 | 3.886 | 0.5132 | Yes |
| 301 | NCAPD2 |  |  | 727 | 3.884 | 0.5146 | Yes |
| 302 | BACE2 |  |  | 728 | 3.883 | 0.5160 | Yes |
| 303 | CAV2 |  |  | 730 | 3.881 | 0.5173 | Yes |
| 304 | CALB2 |  |  | 736 | 3.867 | 0.5182 | Yes |
| 305 | CEP57 |  |  | 739 | 3.862 | 0.5195 | Yes |
| 306 | SERBP1 |  |  | 742 | 3.856 | 0.5207 | Yes |
| 307 | RHCG |  |  | 746 | 3.852 | 0.5218 | Yes |
| 308 | GAPDH |  |  | 747 | 3.851 | 0.5232 | Yes |
| 309 | TFDP1 |  |  | 750 | 3.843 | 0.5244 | Yes |
| 310 | HRB |  |  | 752 | 3.840 | 0.5256 | Yes |
| 311 | CSPG4 |  |  | 756 | 3.835 | 0.5268 | Yes |
| 312 | PIR |  |  | 757 | 3.834 | 0.5281 | Yes |
| 313 | PRDX4 |  |  | 761 | 3.821 | 0.5292 | Yes |
| 314 | UCHL5 |  |  | 765 | 3.814 | 0.5303 | Yes |
| 315 | RIPK2 |  |  | 772 | 3.804 | 0.5312 | Yes |
| 316 | YWHAQ |  |  | 774 | 3.803 | 0.5325 | Yes |
| 317 | HSPC049 |  |  | 777 | 3.801 | 0.5337 | Yes |
| 318 | CEP170 |  |  | 789 | 3.790 | 0.5341 | Yes |
| 319 | FNDC3B |  |  | 797 | 3.785 | 0.5349 | Yes |
| 320 | SLC7A1 |  |  | 798 | 3.782 | 0.5363 | Yes |
| 321 | FOLR1 |  |  | 800 | 3.774 | 0.5375 | Yes |
| 322 | BIN1 |  |  | 804 | 3.772 | 0.5386 | Yes |
| 323 | NPR3 |  |  | 807 | 3.767 | 0.5398 | Yes |
| 324 | WARS |  |  | 810 | 3.765 | 0.5410 | Yes |
| 325 | SOSTDC1 |  |  | 814 | 3.756 | 0.5421 | Yes |
| 326 | MSH2 |  |  | 819 | 3.748 | 0.5431 | Yes |
| 327 | UGP2 |  |  | 822 | 3.745 | 0.5442 | Yes |
| 328 | KLK5 |  |  | 824 | 3.744 | 0.5455 | Yes |
| 329 | TYMS |  |  | 829 | 3.738 | 0.5465 | Yes |
| 330 | SERPINB5 |  |  | 834 | 3.735 | 0.5475 | Yes |
| 331 | SCHIP1 |  |  | 835 | 3.733 | 0.5488 | Yes |
| 332 | KLK7 |  |  | 839 | 3.729 | 0.5499 | Yes |
| 333 | TCF20 |  |  | 846 | 3.712 | 0.5507 | Yes |
| 334 | CHIC2 |  |  | 847 | 3.711 | 0.5520 | Yes |
| 335 | C1ORF38 |  |  | 850 | 3.705 | 0.5532 | Yes |
| 336 | LIPG |  |  | 851 | 3.705 | 0.5545 | Yes |
| 337 | CLDN1 |  |  | 859 | 3.689 | 0.5553 | Yes |
| 338 | CDC2L6 |  |  | 866 | 3.677 | 0.5561 | Yes |
| 339 | ATP5G3 |  |  | 874 | 3.667 | 0.5568 | Yes |
| 340 | RELB |  |  | 875 | 3.666 | 0.5581 | Yes |
| 341 | S100A6 |  |  | 880 | 3.653 | 0.5591 | Yes |
| 342 | PTS |  |  | 884 | 3.648 | 0.5602 | Yes |
| 343 | KIAA0179 |  |  | 885 | 3.648 | 0.5614 | Yes |
| 344 | GDI2 |  |  | 886 | 3.648 | 0.5627 | Yes |
| 345 | RP6-213H19.1 |  |  | 887 | 3.645 | 0.5640 | Yes |
| 346 | RGL1 |  |  | 892 | 3.640 | 0.5650 | Yes |
| 347 | PHGDH |  |  | 893 | 3.636 | 0.5663 | Yes |
| 348 | LY75 |  |  | 905 | 3.630 | 0.5667 | Yes |
| 349 | KIAA1609 |  |  | 908 | 3.625 | 0.5678 | Yes |
| 350 | CA9 |  |  | 909 | 3.623 | 0.5691 | Yes |
| 351 | LAD1 |  |  | 911 | 3.621 | 0.5703 | Yes |
| 352 | RLF |  |  | 914 | 3.618 | 0.5714 | Yes |
| 353 | ATP2C1 |  |  | 915 | 3.618 | 0.5727 | Yes |
| 354 | MRPL15 |  |  | 935 | 3.574 | 0.5725 | Yes |
| 355 | PPARGC1A |  |  | 940 | 3.565 | 0.5734 | Yes |
| 356 | PRIM2A |  |  | 941 | 3.564 | 0.5747 | Yes |
| 357 | PDIA6 |  |  | 953 | 3.547 | 0.5750 | Yes |
| 358 | C21ORF91 |  |  | 956 | 3.543 | 0.5761 | Yes |
| 359 | ATF4 |  |  | 959 | 3.540 | 0.5772 | Yes |
| 360 | SPOCK2 |  |  | 960 | 3.540 | 0.5785 | Yes |
| 361 | PDXK |  |  | 961 | 3.540 | 0.5798 | Yes |
| 362 | CHORDC1 |  |  | 963 | 3.533 | 0.5809 | Yes |
| 363 | MTMR2 |  |  | 965 | 3.531 | 0.5821 | Yes |
| 364 | CKAP4 |  |  | 966 | 3.531 | 0.5834 | Yes |
| 365 | FDX1 |  |  | 969 | 3.527 | 0.5844 | Yes |
| 366 | CHAF1A |  |  | 971 | 3.525 | 0.5856 | Yes |
| 367 | PCDH8 |  |  | 974 | 3.523 | 0.5867 | Yes |
| 368 | ADORA2B |  |  | 976 | 3.519 | 0.5879 | Yes |
| 369 | NUP155 |  |  | 977 | 3.517 | 0.5891 | Yes |
| 370 | HLA-F |  |  | 982 | 3.507 | 0.5900 | Yes |
| 371 | DDX39 |  |  | 989 | 3.498 | 0.5908 | Yes |
| 372 | UBE2C |  |  | 994 | 3.489 | 0.5917 | Yes |
| 373 | INDO |  |  | 995 | 3.489 | 0.5930 | Yes |
| 374 | PLSCR1 |  |  | 1002 | 3.481 | 0.5937 | Yes |
| 375 | HMGN3 |  |  | 1005 | 3.478 | 0.5948 | Yes |
| 376 | RPL39L |  |  | 1009 | 3.473 | 0.5958 | Yes |
| 377 | CCL18 |  |  | 1032 | 3.440 | 0.5952 | Yes |
| 378 | CPA4 |  |  | 1033 | 3.433 | 0.5964 | Yes |
| 379 | SACS |  |  | 1039 | 3.426 | 0.5973 | Yes |
| 380 | RNF138 |  |  | 1042 | 3.421 | 0.5983 | Yes |
| 381 | ADM |  |  | 1046 | 3.419 | 0.5993 | Yes |
| 382 | SNRPA1 |  |  | 1064 | 3.399 | 0.5991 | Yes |
| 383 | DSG2 |  |  | 1068 | 3.393 | 0.6001 | Yes |
| 384 | ETV5 |  |  | 1073 | 3.387 | 0.6010 | Yes |
| 385 | IL6 |  |  | 1081 | 3.378 | 0.6016 | Yes |
| 386 | LSR |  |  | 1082 | 3.377 | 0.6028 | Yes |
| 387 | CD83 |  |  | 1086 | 3.374 | 0.6038 | Yes |
| 388 | FOXD1 |  |  | 1088 | 3.373 | 0.6049 | Yes |
| 389 | PPP2R1B |  |  | 1092 | 3.369 | 0.6058 | Yes |
| 390 | RGS2 |  |  | 1093 | 3.369 | 0.6070 | Yes |
| 391 | NDUFA9 |  |  | 1095 | 3.366 | 0.6081 | Yes |
| 392 | MMP12 |  |  | 1100 | 3.360 | 0.6090 | Yes |
| 393 | DHCR7 |  |  | 1101 | 3.358 | 0.6102 | Yes |
| 394 | TUBB3 |  |  | 1109 | 3.350 | 0.6108 | Yes |
| 395 | SOD2 |  |  | 1111 | 3.347 | 0.6119 | Yes |
| 396 | FBL |  |  | 1116 | 3.344 | 0.6128 | Yes |
| 397 | S100A1 |  |  | 1118 | 3.342 | 0.6139 | Yes |
| 398 | PLS3 |  |  | 1125 | 3.331 | 0.6146 | Yes |
| 399 | LTB |  |  | 1127 | 3.330 | 0.6157 | Yes |
| 400 | HMGB3 |  |  | 1128 | 3.330 | 0.6169 | Yes |
| 401 | KIAA0746 |  |  | 1132 | 3.327 | 0.6178 | Yes |
| 402 | ANXA3 |  |  | 1142 | 3.317 | 0.6183 | Yes |
| 403 | GATA6 |  |  | 1143 | 3.316 | 0.6195 | Yes |
| 404 | CCT4 |  |  | 1148 | 3.309 | 0.6203 | Yes |
| 405 | SLC39A14 |  |  | 1149 | 3.309 | 0.6215 | Yes |
| 406 | PFKP |  |  | 1165 | 3.281 | 0.6214 | Yes |
| 407 | PRRG1 |  |  | 1171 | 3.274 | 0.6222 | Yes |
| 408 | P2RY6 |  |  | 1182 | 3.259 | 0.6226 | Yes |
| 409 | PRG1 |  |  | 1184 | 3.255 | 0.6236 | Yes |
| 410 | RSRC1 |  |  | 1186 | 3.254 | 0.6247 | Yes |
| 411 | PLTP |  |  | 1205 | 3.229 | 0.6244 | Yes |
| 412 | LTBP1 |  |  | 1206 | 3.229 | 0.6255 | Yes |
| 413 | IL1B |  |  | 1211 | 3.226 | 0.6264 | Yes |
| 414 | SRPRB |  |  | 1228 | 3.210 | 0.6262 | Yes |
| 415 | ADAMDEC1 |  |  | 1230 | 3.209 | 0.6273 | Yes |
| 416 | EFS |  |  | 1238 | 3.202 | 0.6279 | Yes |
| 417 | NP |  |  | 1241 | 3.199 | 0.6288 | Yes |
| 418 | DMN |  |  | 1249 | 3.191 | 0.6294 | Yes |
| 419 | ECHDC1 |  |  | 1250 | 3.189 | 0.6305 | Yes |
| 420 | RSU1 |  |  | 1252 | 3.188 | 0.6316 | Yes |
| 421 | CDC2 |  |  | 1259 | 3.182 | 0.6322 | Yes |
| 422 | KRT7 |  |  | 1262 | 3.180 | 0.6332 | Yes |
| 423 | GALNT3 |  |  | 1265 | 3.174 | 0.6342 | Yes |
| 424 | SMYD2 |  |  | 1283 | 3.158 | 0.6339 | Yes |
| 425 | PTPLA |  |  | 1287 | 3.152 | 0.6348 | Yes |
| 426 | PTK7 |  |  | 1289 | 3.149 | 0.6358 | Yes |
| 427 | NUP160 |  |  | 1291 | 3.147 | 0.6369 | Yes |
| 428 | PRKCI |  |  | 1299 | 3.139 | 0.6374 | Yes |
| 429 | FBXO5 |  |  | 1304 | 3.137 | 0.6382 | Yes |
| 430 | HDGF |  |  | 1310 | 3.128 | 0.6389 | Yes |
| 431 | SEMA4D |  |  | 1328 | 3.107 | 0.6387 | Yes |
| 432 | MTRF1L |  |  | 1332 | 3.100 | 0.6395 | Yes |
| 433 | TMSB10 |  |  | 1333 | 3.100 | 0.6406 | Yes |
| 434 | CCL5 |  |  | 1340 | 3.091 | 0.6412 | Yes |
| 435 | ADFP |  |  | 1342 | 3.090 | 0.6422 | Yes |
| 436 | PSMB9 |  |  | 1354 | 3.070 | 0.6425 | Yes |
| 437 | ACTR3B |  |  | 1355 | 3.070 | 0.6435 | Yes |
| 438 | KIF3C |  |  | 1368 | 3.066 | 0.6437 | Yes |
| 439 | TRAF1 |  |  | 1376 | 3.061 | 0.6442 | Yes |
| 440 | CMPK |  |  | 1378 | 3.060 | 0.6452 | Yes |
| 441 | COX4I1 |  |  | 1394 | 3.045 | 0.6451 | Yes |
| 442 | HIF1A |  |  | 1396 | 3.044 | 0.6461 | Yes |
| 443 | QPCT |  |  | 1397 | 3.042 | 0.6472 | Yes |
| 444 | C1ORF112 |  |  | 1410 | 3.032 | 0.6473 | Yes |
| 445 | KLK3 |  |  | 1418 | 3.023 | 0.6478 | Yes |
| 446 | TMEM48 |  |  | 1424 | 3.020 | 0.6484 | Yes |
| 447 | FUT4 |  |  | 1425 | 3.020 | 0.6495 | Yes |
| 448 | S100A10 |  |  | 1435 | 3.014 | 0.6499 | Yes |
| 449 | HSPA14 |  |  | 1437 | 3.012 | 0.6509 | Yes |
| 450 | ADAM19 |  |  | 1440 | 3.010 | 0.6518 | Yes |
| 451 | S100A8 |  |  | 1444 | 3.003 | 0.6526 | Yes |
| 452 | HSD17B12 |  |  | 1449 | 3.000 | 0.6533 | Yes |
| 453 | CENPM |  |  | 1453 | 2.995 | 0.6541 | Yes |
| 454 | PLA2G7 |  |  | 1454 | 2.995 | 0.6552 | Yes |
| 455 | SLC25A37 |  |  | 1455 | 2.995 | 0.6563 | Yes |
| 456 | AQP9 |  |  | 1459 | 2.989 | 0.6571 | Yes |
| 457 | DERA |  |  | 1469 | 2.979 | 0.6574 | Yes |
| 458 | RIMS3 |  |  | 1471 | 2.974 | 0.6584 | Yes |
| 459 | ASB9 |  |  | 1472 | 2.973 | 0.6595 | Yes |
| 460 | TNFRSF1B |  |  | 1474 | 2.970 | 0.6604 | Yes |
| 461 | CLDN10 |  |  | 1485 | 2.957 | 0.6607 | Yes |
| 462 | CKAP2 |  |  | 1492 | 2.951 | 0.6612 | Yes |
| 463 | RCN1 |  |  | 1497 | 2.946 | 0.6620 | Yes |
| 464 | ACN9 |  |  | 1499 | 2.944 | 0.6629 | Yes |
| 465 | CBR1 |  |  | 1503 | 2.940 | 0.6637 | Yes |
| 466 | RGS10 |  |  | 1507 | 2.936 | 0.6645 | Yes |
| 467 | NMI |  |  | 1510 | 2.933 | 0.6654 | Yes |
| 468 | GTPBP4 |  |  | 1514 | 2.931 | 0.6662 | Yes |
| 469 | PDK3 |  |  | 1519 | 2.929 | 0.6669 | Yes |
| 470 | TRAF3IP3 |  |  | 1527 | 2.923 | 0.6674 | Yes |
| 471 | ATAD2 |  |  | 1532 | 2.917 | 0.6681 | Yes |
| 472 | LCP1 |  |  | 1534 | 2.913 | 0.6691 | Yes |
| 473 | RPRM |  |  | 1536 | 2.913 | 0.6700 | Yes |
| 474 | HSPC159 |  |  | 1542 | 2.907 | 0.6707 | Yes |
| 475 | GMPS |  |  | 1543 | 2.907 | 0.6717 | Yes |
| 476 | TMCC2 |  |  | 1549 | 2.904 | 0.6723 | Yes |
| 477 | IL1R2 |  |  | 1550 | 2.903 | 0.6733 | Yes |
| 478 | LAMC2 |  |  | 1554 | 2.899 | 0.6741 | Yes |
| 479 | CUGBP2 |  |  | 1556 | 2.898 | 0.6751 | Yes |
| 480 | ASS1 |  |  | 1558 | 2.895 | 0.6760 | Yes |
| 481 | SDCBP |  |  | 1573 | 2.875 | 0.6759 | Yes |
| 482 | BYSL |  |  | 1577 | 2.872 | 0.6767 | Yes |
| 483 | SSRP1 |  |  | 1591 | 2.860 | 0.6767 | Yes |
| 484 | LOC56902 |  |  | 1605 | 2.845 | 0.6766 | Yes |
| 485 | PITRM1 |  |  | 1607 | 2.843 | 0.6776 | Yes |
| 486 | GATAD2A |  |  | 1615 | 2.834 | 0.6780 | Yes |
| 487 | CCNC |  |  | 1622 | 2.826 | 0.6785 | Yes |
| 488 | TGM1 |  |  | 1630 | 2.820 | 0.6790 | Yes |
| 489 | ILF2 |  |  | 1644 | 2.807 | 0.6789 | Yes |
| 490 | SLC11A1 |  |  | 1650 | 2.802 | 0.6795 | Yes |
| 491 | RPP38 |  |  | 1652 | 2.799 | 0.6804 | Yes |
| 492 | GYPC |  |  | 1663 | 2.791 | 0.6806 | Yes |
| 493 | CCL2 |  |  | 1664 | 2.791 | 0.6816 | Yes |
| 494 | TGFBI |  |  | 1668 | 2.786 | 0.6823 | Yes |
| 495 | MRPL11 |  |  | 1693 | 2.764 | 0.6814 | Yes |
| 496 | LCP2 |  |  | 1694 | 2.764 | 0.6824 | Yes |
| 497 | TDO2 |  |  | 1700 | 2.761 | 0.6830 | Yes |
| 498 | NEK2 |  |  | 1703 | 2.758 | 0.6838 | Yes |
| 499 | PKMYT1 |  |  | 1715 | 2.753 | 0.6839 | Yes |
| 500 | BIRC3 |  |  | 1726 | 2.745 | 0.6840 | Yes |
| 501 | HK3 |  |  | 1745 | 2.729 | 0.6836 | Yes |
| 502 | RASSF2 |  |  | 1746 | 2.729 | 0.6845 | Yes |
| 503 | ELAVL1 |  |  | 1751 | 2.727 | 0.6852 | Yes |
| 504 | DNMT3A |  |  | 1755 | 2.722 | 0.6859 | Yes |
| 505 | GAPDHS |  |  | 1763 | 2.717 | 0.6863 | Yes |
| 506 | SFN |  |  | 1767 | 2.716 | 0.6870 | Yes |
| 507 | CD14 |  |  | 1768 | 2.716 | 0.6880 | Yes |
| 508 | SLC2A5 |  |  | 1776 | 2.711 | 0.6884 | Yes |
| 509 | KIAA0922 |  |  | 1784 | 2.705 | 0.6888 | Yes |
| 510 | CLCN4 |  |  | 1791 | 2.702 | 0.6893 | Yes |
| 511 | CMAS |  |  | 1800 | 2.690 | 0.6896 | Yes |
| 512 | TMEM51 |  |  | 1801 | 2.689 | 0.6905 | Yes |
| 513 | BCL2A1 |  |  | 1804 | 2.688 | 0.6913 | Yes |
| 514 | PTDSS1 |  |  | 1806 | 2.686 | 0.6922 | Yes |
| 515 | SCARA3 |  |  | 1813 | 2.683 | 0.6927 | Yes |
| 516 | ARFGAP3 |  |  | 1837 | 2.664 | 0.6918 | Yes |
| 517 | KLK6 |  |  | 1839 | 2.657 | 0.6926 | Yes |
| 518 | FNBP1 |  |  | 1841 | 2.656 | 0.6935 | Yes |
| 519 | PITPNA |  |  | 1845 | 2.651 | 0.6942 | Yes |
| 520 | TBPL1 |  |  | 1848 | 2.650 | 0.6950 | Yes |
| 521 | VCAM1 |  |  | 1849 | 2.649 | 0.6959 | Yes |
| 522 | MTF2 |  |  | 1858 | 2.643 | 0.6962 | Yes |
| 523 | SYK |  |  | 1859 | 2.641 | 0.6971 | Yes |
| 524 | USP10 |  |  | 1872 | 2.629 | 0.6971 | Yes |
| 525 | CXCR6 |  |  | 1879 | 2.624 | 0.6976 | Yes |
| 526 | HECA |  |  | 1885 | 2.618 | 0.6981 | Yes |
| 527 | LSM7 |  |  | 1887 | 2.617 | 0.6989 | Yes |
| 528 | MMP9 |  |  | 1891 | 2.616 | 0.6996 | Yes |
| 529 | FLNA |  |  | 1895 | 2.609 | 0.7003 | Yes |
| 530 | PDE4B |  |  | 1897 | 2.609 | 0.7012 | Yes |
| 531 | FLJ10292 |  |  | 1898 | 2.608 | 0.7021 | Yes |
| 532 | CHD7 |  |  | 1909 | 2.601 | 0.7022 | Yes |
| 533 | VSIG4 |  |  | 1920 | 2.591 | 0.7023 | Yes |
| 534 | KLK10 |  |  | 1929 | 2.588 | 0.7026 | Yes |
| 535 | CDK6 |  |  | 1932 | 2.584 | 0.7033 | Yes |
| 536 | TNFAIP2 |  |  | 1940 | 2.580 | 0.7037 | Yes |
| 537 | CCL4 |  |  | 1943 | 2.576 | 0.7045 | Yes |
| 538 | RIOK3 |  |  | 1951 | 2.571 | 0.7048 | Yes |
| 539 | PSMA4 |  |  | 1960 | 2.564 | 0.7051 | Yes |
| 540 | HYOU1 |  |  | 1971 | 2.555 | 0.7052 | Yes |
| 541 | CRABP1 |  |  | 1975 | 2.553 | 0.7058 | Yes |
| 542 | CRLF3 |  |  | 1987 | 2.539 | 0.7059 | Yes |
| 543 | HCK |  |  | 1992 | 2.537 | 0.7064 | Yes |
| 544 | RASGRP2 |  |  | 2003 | 2.527 | 0.7065 | Yes |
| 545 | EPRS |  |  | 2004 | 2.527 | 0.7074 | Yes |
| 546 | ECT2 |  |  | 2026 | 2.509 | 0.7066 | Yes |
| 547 | GDF5 |  |  | 2038 | 2.496 | 0.7066 | Yes |
| 548 | SLC2A1 |  |  | 2043 | 2.490 | 0.7072 | Yes |
| 549 | WTAP |  |  | 2044 | 2.490 | 0.7081 | Yes |
| 550 | RPN1 |  |  | 2053 | 2.479 | 0.7083 | Yes |
| 551 | AIM2 |  |  | 2063 | 2.474 | 0.7085 | Yes |
| 552 | TCF7 |  |  | 2067 | 2.473 | 0.7091 | Yes |
| 553 | GNPTAB |  |  | 2079 | 2.464 | 0.7091 | Yes |
| 554 | SAMD4A |  |  | 2082 | 2.462 | 0.7098 | Yes |
| 555 | DNM1L |  |  | 2087 | 2.459 | 0.7104 | Yes |
| 556 | EML4 |  |  | 2092 | 2.457 | 0.7109 | Yes |
| 557 | KCNJ15 |  |  | 2104 | 2.449 | 0.7109 | Yes |
| 558 | H2AFZ |  |  | 2105 | 2.447 | 0.7118 | Yes |
| 559 | VIM |  |  | 2118 | 2.443 | 0.7117 | Yes |
| 560 | BPI |  |  | 2121 | 2.442 | 0.7124 | Yes |
| 561 | GPR109B |  |  | 2126 | 2.438 | 0.7129 | Yes |
| 562 | PSMB4 |  |  | 2131 | 2.433 | 0.7135 | Yes |
| 563 | SOD3 |  |  | 2135 | 2.429 | 0.7141 | Yes |
| 564 | SLC31A1 |  |  | 2149 | 2.417 | 0.7139 | Yes |
| 565 | CSTA |  |  | 2157 | 2.414 | 0.7142 | Yes |
| 566 | CNGA1 |  |  | 2164 | 2.410 | 0.7146 | Yes |
| 567 | C1ORF106 |  |  | 2166 | 2.409 | 0.7153 | Yes |
| 568 | TLX3 |  |  | 2202 | 2.386 | 0.7134 | Yes |
| 569 | PLEK |  |  | 2206 | 2.384 | 0.7140 | Yes |
| 570 | SEC61A1 |  |  | 2208 | 2.383 | 0.7147 | Yes |
| 571 | TXN |  |  | 2214 | 2.379 | 0.7152 | Yes |
| 572 | NRAS |  |  | 2224 | 2.371 | 0.7153 | Yes |
| 573 | SFRS7 |  |  | 2226 | 2.370 | 0.7161 | Yes |
| 574 | MMP1 |  |  | 2251 | 2.350 | 0.7150 | Yes |
| 575 | SREBF2 |  |  | 2252 | 2.349 | 0.7158 | Yes |
| 576 | USP13 |  |  | 2253 | 2.349 | 0.7166 | Yes |
| 577 | DUSP2 |  |  | 2261 | 2.343 | 0.7169 | Yes |
| 578 | GCLM |  |  | 2267 | 2.339 | 0.7173 | Yes |
| 579 | C1S |  |  | 2270 | 2.337 | 0.7180 | Yes |
| 580 | LIMK2 |  |  | 2272 | 2.335 | 0.7188 | Yes |
| 581 | CLEC4A |  |  | 2294 | 2.327 | 0.7179 | Yes |
| 582 | HAAO |  |  | 2297 | 2.323 | 0.7186 | Yes |
| 583 | FYB |  |  | 2314 | 2.313 | 0.7181 | Yes |
| 584 | TRIT1 |  |  | 2319 | 2.311 | 0.7186 | Yes |
| 585 | DUSP9 |  |  | 2335 | 2.300 | 0.7182 | Yes |
| 586 | ADAM8 |  |  | 2347 | 2.288 | 0.7181 | Yes |
| 587 | SSR1 |  |  | 2351 | 2.285 | 0.7187 | Yes |
| 588 | SOAT1 |  |  | 2362 | 2.281 | 0.7187 | Yes |
| 589 | LY6D |  |  | 2372 | 2.273 | 0.7188 | Yes |
| 590 | DR1 |  |  | 2383 | 2.269 | 0.7188 | Yes |
| 591 | EIF2C2 |  |  | 2384 | 2.269 | 0.7196 | Yes |
| 592 | CCT5 |  |  | 2398 | 2.262 | 0.7194 | Yes |
| 593 | TIA1 |  |  | 2434 | 2.233 | 0.7173 | Yes |
| 594 | DBN1 |  |  | 2438 | 2.229 | 0.7179 | Yes |
| 595 | GLIPR1 |  |  | 2439 | 2.229 | 0.7187 | Yes |
| 596 | PGM3 |  |  | 2445 | 2.225 | 0.7191 | Yes |
| 597 | NDRG1 |  |  | 2463 | 2.218 | 0.7185 | Yes |
| 598 | EPHA4 |  |  | 2467 | 2.218 | 0.7190 | Yes |
| 599 | ACAT2 |  |  | 2472 | 2.215 | 0.7195 | Yes |
| 600 | EBI2 |  |  | 2488 | 2.204 | 0.7191 | Yes |
| 601 | CARHSP1 |  |  | 2492 | 2.203 | 0.7196 | Yes |
| 602 | AIF1 |  |  | 2499 | 2.199 | 0.7199 | Yes |
| 603 | RAB7L1 |  |  | 2511 | 2.194 | 0.7198 | Yes |
| 604 | CDT1 |  |  | 2522 | 2.190 | 0.7198 | Yes |
| 605 | TANK |  |  | 2530 | 2.184 | 0.7200 | Yes |
| 606 | RRBP1 |  |  | 2538 | 2.179 | 0.7202 | Yes |
| 607 | ITGB2 |  |  | 2539 | 2.179 | 0.7210 | Yes |
| 608 | NARF |  |  | 2544 | 2.178 | 0.7214 | Yes |
| 609 | C1QB |  |  | 2564 | 2.166 | 0.7207 | Yes |
| 610 | CYB5R2 |  |  | 2587 | 2.154 | 0.7197 | Yes |
| 611 | HEBP2 |  |  | 2607 | 2.142 | 0.7189 | Yes |
| 612 | PPA1 |  |  | 2614 | 2.138 | 0.7192 | Yes |
| 613 | LEREPO4 |  |  | 2620 | 2.135 | 0.7195 | Yes |
| 614 | PTPRC |  |  | 2622 | 2.133 | 0.7202 | Yes |
| 615 | CORO1A |  |  | 2626 | 2.131 | 0.7207 | Yes |
| 616 | GZMA |  |  | 2627 | 2.130 | 0.7215 | Yes |
| 617 | ACTL6A |  |  | 2632 | 2.128 | 0.7219 | Yes |
| 618 | CASP1 |  |  | 2645 | 2.120 | 0.7217 | Yes |
| 619 | POLA2 |  |  | 2649 | 2.117 | 0.7222 | Yes |
| 620 | BNIP3 |  |  | 2662 | 2.111 | 0.7220 | Yes |
| 621 | C14ORF58 |  |  | 2666 | 2.108 | 0.7225 | Yes |
| 622 | PLAC8 |  |  | 2668 | 2.107 | 0.7232 | Yes |
| 623 | LXN |  |  | 2669 | 2.106 | 0.7239 | Yes |
| 624 | KRT23 |  |  | 2670 | 2.106 | 0.7247 | Yes |
| 625 | SEC13L1 |  |  | 2697 | 2.093 | 0.7233 | No |
| 626 | SLA |  |  | 2700 | 2.092 | 0.7239 | No |
| 627 | INPP5D |  |  | 2716 | 2.081 | 0.7234 | No |
| 628 | CYBB |  |  | 2718 | 2.080 | 0.7241 | No |
| 629 | PSMD2 |  |  | 2759 | 2.063 | 0.7216 | No |
| 630 | GRAMD3 |  |  | 2766 | 2.060 | 0.7219 | No |
| 631 | SOX9 |  |  | 2768 | 2.058 | 0.7225 | No |
| 632 | GIT2 |  |  | 2773 | 2.057 | 0.7229 | No |
| 633 | ITGB4 |  |  | 2781 | 2.051 | 0.7231 | No |
| 634 | AIM1 |  |  | 2807 | 2.037 | 0.7218 | No |
| 635 | EIF5A |  |  | 2812 | 2.033 | 0.7222 | No |
| 636 | FADS1 |  |  | 2837 | 2.021 | 0.7210 | No |
| 637 | CYP27A1 |  |  | 2849 | 2.014 | 0.7208 | No |
| 638 | POU2AF1 |  |  | 2861 | 2.006 | 0.7207 | No |
| 639 | CNN3 |  |  | 2865 | 2.004 | 0.7211 | No |
| 640 | FXYD5 |  |  | 2892 | 1.990 | 0.7197 | No |
| 641 | KCNK1 |  |  | 2906 | 1.983 | 0.7194 | No |
| 642 | HLA-DQB1 |  |  | 2913 | 1.980 | 0.7196 | No |
| 643 | VRK2 |  |  | 2966 | 1.952 | 0.7161 | No |
| 644 | TLR2 |  |  | 2969 | 1.951 | 0.7167 | No |
| 645 | ATP6V0A2 |  |  | 2970 | 1.948 | 0.7174 | No |
| 646 | DENND3 |  |  | 2982 | 1.938 | 0.7172 | No |
| 647 | HTATIP2 |  |  | 2993 | 1.933 | 0.7171 | No |
| 648 | IDH2 |  |  | 3005 | 1.929 | 0.7169 | No |
| 649 | ETV7 |  |  | 3009 | 1.926 | 0.7173 | No |
| 650 | HEATR1 |  |  | 3012 | 1.926 | 0.7178 | No |
| 651 | DAPP1 |  |  | 3014 | 1.926 | 0.7184 | No |
| 652 | DNMT3B |  |  | 3016 | 1.925 | 0.7190 | No |
| 653 | PWP2H |  |  | 3018 | 1.924 | 0.7196 | No |
| 654 | NFKBIE |  |  | 3034 | 1.916 | 0.7191 | No |
| 655 | KYNU |  |  | 3045 | 1.911 | 0.7190 | No |
| 656 | SPEG |  |  | 3052 | 1.908 | 0.7192 | No |
| 657 | ORM1 |  |  | 3054 | 1.906 | 0.7198 | No |
| 658 | CXCR4 |  |  | 3062 | 1.902 | 0.7199 | No |
| 659 | PGM1 |  |  | 3083 | 1.893 | 0.7189 | No |
| 660 | SELE |  |  | 3084 | 1.892 | 0.7196 | No |
| 661 | SLC35C1 |  |  | 3100 | 1.887 | 0.7191 | No |
| 662 | MX2 |  |  | 3118 | 1.881 | 0.7184 | No |
| 663 | NOS3 |  |  | 3135 | 1.873 | 0.7178 | No |
| 664 | IL7R |  |  | 3145 | 1.868 | 0.7177 | No |
| 665 | CDKN2C |  |  | 3148 | 1.866 | 0.7182 | No |
| 666 | NCKAP1L |  |  | 3194 | 1.845 | 0.7152 | No |
| 667 | GLRX2 |  |  | 3196 | 1.841 | 0.7158 | No |
| 668 | FCGR1A |  |  | 3199 | 1.840 | 0.7163 | No |
| 669 | RTCD1 |  |  | 3207 | 1.836 | 0.7164 | No |
| 670 | S100A9 |  |  | 3221 | 1.828 | 0.7160 | No |
| 671 | SEC61G |  |  | 3232 | 1.823 | 0.7158 | No |
| 672 | C18ORF10 |  |  | 3263 | 1.810 | 0.7141 | No |
| 673 | CD300A |  |  | 3267 | 1.809 | 0.7145 | No |
| 674 | TCN2 |  |  | 3279 | 1.803 | 0.7142 | No |
| 675 | ZNF313 |  |  | 3329 | 1.781 | 0.7109 | No |
| 676 | ENY2 |  |  | 3351 | 1.772 | 0.7099 | No |
| 677 | KCTD9 |  |  | 3355 | 1.768 | 0.7103 | No |
| 678 | MAP3K5 |  |  | 3368 | 1.764 | 0.7099 | No |
| 679 | PFKL |  |  | 3387 | 1.756 | 0.7091 | No |
| 680 | MAPK6 |  |  | 3396 | 1.751 | 0.7091 | No |
| 681 | ATF3 |  |  | 3455 | 1.727 | 0.7050 | No |
| 682 | CD180 |  |  | 3469 | 1.723 | 0.7046 | No |
| 683 | TOPBP1 |  |  | 3475 | 1.722 | 0.7048 | No |
| 684 | CD86 |  |  | 3484 | 1.716 | 0.7048 | No |
| 685 | TMEM45A |  |  | 3492 | 1.713 | 0.7048 | No |
| 686 | TBCCD1 |  |  | 3517 | 1.699 | 0.7035 | No |
| 687 | IFI30 |  |  | 3555 | 1.684 | 0.7011 | No |
| 688 | CD3G |  |  | 3570 | 1.676 | 0.7006 | No |
| 689 | PHACTR2 |  |  | 3606 | 1.660 | 0.6984 | No |
| 690 | CHI3L2 |  |  | 3623 | 1.651 | 0.6977 | No |
| 691 | CD44 |  |  | 3638 | 1.645 | 0.6971 | No |
| 692 | CYBA |  |  | 3650 | 1.640 | 0.6968 | No |
| 693 | IL2RB |  |  | 3652 | 1.639 | 0.6973 | No |
| 694 | MLSTD1 |  |  | 3662 | 1.637 | 0.6972 | No |
| 695 | ITSN2 |  |  | 3670 | 1.632 | 0.6972 | No |
| 696 | IFI44 |  |  | 3696 | 1.621 | 0.6958 | No |
| 697 | DNAJC15 |  |  | 3697 | 1.621 | 0.6964 | No |
| 698 | ZNF267 |  |  | 3725 | 1.614 | 0.6948 | No |
| 699 | ETS1 |  |  | 3729 | 1.611 | 0.6951 | No |
| 700 | CD3D |  |  | 3733 | 1.611 | 0.6954 | No |
| 701 | STEAP3 |  |  | 3738 | 1.609 | 0.6957 | No |
| 702 | CNIH4 |  |  | 3748 | 1.606 | 0.6955 | No |
| 703 | LAMP2 |  |  | 3775 | 1.596 | 0.6940 | No |
| 704 | GMFG |  |  | 3780 | 1.593 | 0.6942 | No |
| 705 | SLC1A3 |  |  | 3805 | 1.586 | 0.6929 | No |
| 706 | CPVL |  |  | 3832 | 1.578 | 0.6914 | No |
| 707 | IFNGR1 |  |  | 3857 | 1.569 | 0.6900 | No |
| 708 | PDCD10 |  |  | 3874 | 1.564 | 0.6893 | No |
| 709 | SLC7A7 |  |  | 3876 | 1.563 | 0.6897 | No |
| 710 | FAM49B |  |  | 3884 | 1.558 | 0.6897 | No |
| 711 | PRPF18 |  |  | 3904 | 1.551 | 0.6887 | No |
| 712 | VEGF |  |  | 3911 | 1.549 | 0.6888 | No |
| 713 | TMEM40 |  |  | 3928 | 1.543 | 0.6881 | No |
| 714 | GSTA3 |  |  | 3934 | 1.540 | 0.6882 | No |
| 715 | PRAME |  |  | 3945 | 1.537 | 0.6880 | No |
| 716 | FAM98A |  |  | 3991 | 1.515 | 0.6849 | No |
| 717 | GIMAP5 |  |  | 4022 | 1.503 | 0.6830 | No |
| 718 | PLEKHB1 |  |  | 4033 | 1.500 | 0.6827 | No |
| 719 | DRAM |  |  | 4060 | 1.489 | 0.6812 | No |
| 720 | KLRD1 |  |  | 4067 | 1.486 | 0.6812 | No |
| 721 | SELL |  |  | 4075 | 1.483 | 0.6812 | No |
| 722 | CENTD1 |  |  | 4076 | 1.482 | 0.6817 | No |
| 723 | DTX2 |  |  | 4077 | 1.482 | 0.6822 | No |
| 724 | CENTG1 |  |  | 4078 | 1.481 | 0.6828 | No |
| 725 | TPRKB |  |  | 4092 | 1.476 | 0.6822 | No |
| 726 | RACGAP1 |  |  | 4097 | 1.475 | 0.6824 | No |
| 727 | KDELR3 |  |  | 4101 | 1.472 | 0.6827 | No |
| 728 | PTBP2 |  |  | 4123 | 1.463 | 0.6816 | No |
| 729 | LGALS9 |  |  | 4151 | 1.452 | 0.6799 | No |
| 730 | CD53 |  |  | 4158 | 1.449 | 0.6799 | No |
| 731 | CASP4 |  |  | 4175 | 1.442 | 0.6792 | No |
| 732 | BNIP2 |  |  | 4214 | 1.425 | 0.6766 | No |
| 733 | UFD1L |  |  | 4248 | 1.414 | 0.6745 | No |
| 734 | PSCD4 |  |  | 4250 | 1.413 | 0.6749 | No |
| 735 | SLC4A1 |  |  | 4284 | 1.399 | 0.6727 | No |
| 736 | NCF2 |  |  | 4303 | 1.390 | 0.6718 | No |
| 737 | RPS6KA4 |  |  | 4307 | 1.389 | 0.6720 | No |
| 738 | FMNL1 |  |  | 4315 | 1.384 | 0.6720 | No |
| 739 | RIT1 |  |  | 4352 | 1.372 | 0.6696 | No |
| 740 | PDZK1IP1 |  |  | 4364 | 1.369 | 0.6692 | No |
| 741 | PTPRCAP |  |  | 4392 | 1.359 | 0.6675 | No |
| 742 | IRF5 |  |  | 4410 | 1.351 | 0.6666 | No |
| 743 | RFTN1 |  |  | 4455 | 1.333 | 0.6635 | No |
| 744 | PSCD1 |  |  | 4539 | 1.306 | 0.6573 | No |
| 745 | SLC39A8 |  |  | 4540 | 1.306 | 0.6578 | No |
| 746 | EIF2B3 |  |  | 4557 | 1.300 | 0.6570 | No |
| 747 | S100A11 |  |  | 4606 | 1.284 | 0.6536 | No |
| 748 | SIRPG |  |  | 4616 | 1.281 | 0.6533 | No |
| 749 | STAT1 |  |  | 4617 | 1.280 | 0.6538 | No |
| 750 | WDR62 |  |  | 4646 | 1.270 | 0.6520 | No |
| 751 | C1ORF41 |  |  | 4672 | 1.262 | 0.6504 | No |
| 752 | ACOT7 |  |  | 4673 | 1.261 | 0.6509 | No |
| 753 | PALMD |  |  | 4722 | 1.246 | 0.6474 | No |
| 754 | INTS7 |  |  | 4743 | 1.239 | 0.6463 | No |
| 755 | HIST1H1B |  |  | 4744 | 1.238 | 0.6467 | No |
| 756 | SLAMF7 |  |  | 4769 | 1.231 | 0.6452 | No |
| 757 | TRIM22 |  |  | 4808 | 1.220 | 0.6426 | No |
| 758 | SNN |  |  | 4809 | 1.220 | 0.6430 | No |
| 759 | DNAJA2 |  |  | 4817 | 1.217 | 0.6429 | No |
| 760 | CYP1B1 |  |  | 4821 | 1.215 | 0.6431 | No |
| 761 | FANCL |  |  | 4824 | 1.213 | 0.6434 | No |
| 762 | GARS |  |  | 4828 | 1.211 | 0.6436 | No |
| 763 | CEP192 |  |  | 4874 | 1.197 | 0.6404 | No |
| 764 | VPREB3 |  |  | 4876 | 1.197 | 0.6407 | No |
| 765 | PLA2G2A |  |  | 4925 | 1.182 | 0.6373 | No |
| 766 | HCLS1 |  |  | 4927 | 1.181 | 0.6376 | No |
| 767 | CD48 |  |  | 4940 | 1.175 | 0.6371 | No |
| 768 | STK10 |  |  | 4944 | 1.174 | 0.6372 | No |
| 769 | HN1 |  |  | 4953 | 1.171 | 0.6370 | No |
| 770 | IGSF6 |  |  | 4987 | 1.160 | 0.6348 | No |
| 771 | GPR126 |  |  | 4994 | 1.158 | 0.6347 | No |
| 772 | CALML5 |  |  | 4996 | 1.157 | 0.6350 | No |
| 773 | IGLV6-57 |  |  | 5044 | 1.138 | 0.6317 | No |
| 774 | PTPLB |  |  | 5075 | 1.125 | 0.6297 | No |
| 775 | FLI1 |  |  | 5103 | 1.114 | 0.6279 | No |
| 776 | C11ORF73 |  |  | 5141 | 1.101 | 0.6253 | No |
| 777 | PIM2 |  |  | 5147 | 1.099 | 0.6253 | No |
| 778 | STAT4 |  |  | 5170 | 1.091 | 0.6239 | No |
| 779 | FLJ10154 |  |  | 5200 | 1.081 | 0.6220 | No |
| 780 | GIMAP4 |  |  | 5218 | 1.076 | 0.6210 | No |
| 781 | LAPTM5 |  |  | 5239 | 1.068 | 0.6198 | No |
| 782 | RASIP1 |  |  | 5251 | 1.063 | 0.6193 | No |
| 783 | ESRRA |  |  | 5265 | 1.060 | 0.6186 | No |
| 784 | EED |  |  | 5267 | 1.060 | 0.6189 | No |
| 785 | EVI2B |  |  | 5320 | 1.038 | 0.6151 | No |
| 786 | CD52 |  |  | 5341 | 1.031 | 0.6138 | No |
| 787 | TUBB4 |  |  | 5352 | 1.025 | 0.6134 | No |
| 788 | CCR2 |  |  | 5356 | 1.025 | 0.6135 | No |
| 789 | PITPNM1 |  |  | 5370 | 1.022 | 0.6128 | No |
| 790 | RYK |  |  | 5371 | 1.022 | 0.6132 | No |
| 791 | ECGF1 |  |  | 5392 | 1.016 | 0.6119 | No |
| 792 | HIST1H4B |  |  | 5419 | 1.003 | 0.6102 | No |
| 793 | CRLF1 |  |  | 5434 | 1.000 | 0.6094 | No |
| 794 | SAMHD1 |  |  | 5458 | 0.990 | 0.6080 | No |
| 795 | PTGDS |  |  | 5528 | 0.965 | 0.6028 | No |
| 796 | IGLL1 |  |  | 5555 | 0.957 | 0.6010 | No |
| 797 | KIAA0103 |  |  | 5618 | 0.939 | 0.5964 | No |
| 798 | PPP2R3B |  |  | 5635 | 0.935 | 0.5954 | No |
| 799 | BIRC2 |  |  | 5640 | 0.933 | 0.5954 | No |
| 800 | RNASE1 |  |  | 5702 | 0.910 | 0.5908 | No |
| 801 | PTTG1IP |  |  | 5711 | 0.907 | 0.5905 | No |
| 802 | CCL19 |  |  | 5720 | 0.905 | 0.5902 | No |
| 803 | PEX13 |  |  | 5731 | 0.902 | 0.5897 | No |
| 804 | ISG20 |  |  | 5742 | 0.898 | 0.5892 | No |
| 805 | GCH1 |  |  | 5810 | 0.875 | 0.5842 | No |
| 806 | PGK1 |  |  | 5836 | 0.865 | 0.5825 | No |
| 807 | FASLG |  |  | 5917 | 0.842 | 0.5763 | No |
| 808 | GBP2 |  |  | 5953 | 0.831 | 0.5738 | No |
| 809 | BRDG1 |  |  | 6025 | 0.801 | 0.5684 | No |
| 810 | NUDT21 |  |  | 6037 | 0.798 | 0.5678 | No |
| 811 | TYROBP |  |  | 6079 | 0.787 | 0.5648 | No |
| 812 | ACAA2 |  |  | 6109 | 0.780 | 0.5627 | No |
| 813 | KIF21B |  |  | 6113 | 0.780 | 0.5628 | No |
| 814 | SLC16A3 |  |  | 6141 | 0.771 | 0.5609 | No |
| 815 | ALG6 |  |  | 6157 | 0.763 | 0.5600 | No |
| 816 | LAP3 |  |  | 6186 | 0.754 | 0.5580 | No |
| 817 | CCDC109B |  |  | 6228 | 0.738 | 0.5549 | No |
| 818 | FADS2 |  |  | 6236 | 0.735 | 0.5546 | No |
| 819 | FGL2 |  |  | 6238 | 0.734 | 0.5548 | No |
| 820 | RAB32 |  |  | 6275 | 0.724 | 0.5522 | No |
| 821 | LPXN |  |  | 6310 | 0.713 | 0.5497 | No |
| 822 | CCR7 |  |  | 6360 | 0.698 | 0.5460 | No |
| 823 | PSPH |  |  | 6375 | 0.692 | 0.5451 | No |
| 824 | AARS |  |  | 6422 | 0.675 | 0.5417 | No |
| 825 | GPNMB |  |  | 6438 | 0.671 | 0.5407 | No |
| 826 | EDN2 |  |  | 6495 | 0.652 | 0.5365 | No |
| 827 | DEGS1 |  |  | 6528 | 0.640 | 0.5341 | No |
| 828 | CD247 |  |  | 6551 | 0.634 | 0.5326 | No |
| 829 | WDSOF1 |  |  | 6566 | 0.628 | 0.5317 | No |
| 830 | TRIP6 |  |  | 6573 | 0.627 | 0.5314 | No |
| 831 | TMEM140 |  |  | 6592 | 0.624 | 0.5302 | No |
| 832 | IL10RA |  |  | 6598 | 0.621 | 0.5300 | No |
| 833 | FCHO1 |  |  | 6709 | 0.583 | 0.5214 | No |
| 834 | CXCR3 |  |  | 6719 | 0.581 | 0.5209 | No |
| 835 | USP18 |  |  | 6788 | 0.556 | 0.5156 | No |
| 836 | CD2 |  |  | 6802 | 0.549 | 0.5148 | No |
| 837 | SIT1 |  |  | 6819 | 0.545 | 0.5137 | No |
| 838 | CD69 |  |  | 6909 | 0.516 | 0.5067 | No |
| 839 | CLDN4 |  |  | 6946 | 0.504 | 0.5040 | No |
| 840 | CBR3 |  |  | 7039 | 0.473 | 0.4968 | No |
| 841 | CD74 |  |  | 7061 | 0.463 | 0.4952 | No |
| 842 | DOCK2 |  |  | 7068 | 0.462 | 0.4949 | No |
| 843 | DKK1 |  |  | 7386 | 0.349 | 0.4696 | No |
| 844 | MAP3K14 |  |  | 7392 | 0.347 | 0.4693 | No |
| 845 | FAM46A |  |  | 7417 | 0.339 | 0.4675 | No |
| 846 | RSAD2 |  |  | 7548 | 0.291 | 0.4572 | No |
| 847 | ADAMTS7 |  |  | 7622 | 0.261 | 0.4514 | No |
| 848 | DIAPH3 |  |  | 7628 | 0.259 | 0.4511 | No |
| 849 | KIF1B |  |  | 7638 | 0.255 | 0.4505 | No |
| 850 | OAS2 |  |  | 7687 | 0.236 | 0.4467 | No |
| 851 | ENAH |  |  | 7797 | 0.190 | 0.4380 | No |
| 852 | TIMP2 |  |  | 8021 | 0.096 | 0.4201 | No |
| 853 | FUS |  |  | 8367 | -0.024 | 0.3924 | No |
| 854 | CDC34 |  |  | 8499 | -0.076 | 0.3820 | No |
| 855 | PDCD1LG2 |  |  | 8545 | -0.101 | 0.3784 | No |
| 856 | HIST1H1E |  |  | 8561 | -0.106 | 0.3772 | No |
| 857 | KLF6 |  |  | 8882 | -0.249 | 0.3516 | No |
| 858 | COPG |  |  | 9202 | -0.407 | 0.3261 | No |
| 859 | GALK1 |  |  | 9378 | -0.495 | 0.3123 | No |
| 860 | SDC1 |  |  | 9416 | -0.521 | 0.3095 | No |
| 861 | OSBPL7 |  |  | 9781 | -0.713 | 0.2805 | No |
| 862 | DDIT3 |  |  | 9789 | -0.718 | 0.2802 | No |
| 863 | EGR2 |  |  | 9996 | -0.839 | 0.2639 | No |
| 864 | SLC15A3 |  |  | 10011 | -0.853 | 0.2631 | No |
| 865 | CAPG |  |  | 10021 | -0.858 | 0.2627 | No |
| 866 | CYR61 |  |  | 10171 | -0.940 | 0.2511 | No |
| 867 | SUV420H1 |  |  | 13088 | -4.780 | 0.0186 | No |
Table: GSEA details [plain text format]

  

Fig 2: BRCA\_ER\_NEG: Random ES distribution      
 Gene set null distribution of ES for **BRCA\_ER\_NEG**
